# GPs' Perceptions of Cardiovascular Risk and Views on Patient Compliance: A Qualitative Interview Study

**DOI:** 10.1155/2015/214146

**Published:** 2015-10-08

**Authors:** Benedicte Lind Barfoed, Dorte Ejg Jarbøl, Maja Skov Paulsen, Palle Mark Christensen, Peder Andreas Halvorsen, Jesper Bo Nielsen, Jens Søndergaard

**Affiliations:** ^1^Research Unit for General Practice, University of Southern Denmark, JB Winsløws Vej 9A, 5000 Odense C, Denmark; ^2^Danish Quality Unit for General Practice, JB Winsløws Vej 9A, 5000 Odense C, Denmark; ^3^General Practitioners Lærkevej, Lærkevej 14, 5450 Otterup, Denmark; ^4^Department of Community Medicine, University of Tromsø, The Arctic University of Norway, 9037 Tromsø, Norway

## Abstract

*Objective*. General practitioners' (GPs') perception of risk is a cornerstone of preventive care. The aims of this interview study were to explore GPs' professional and personal attitudes and experiences regarding treatment with lipid-lowering drugs and their views on patient compliance. *Methods*. The material was drawn from semistructured qualitative interviews. We sampled GPs purposively from ten selected practices, ensuring diversity of demographic, professional, and personal characteristics. The GPs were encouraged to describe examples from their own practices and reflect on them and were informed that the focus was their personal attitudes and experiences. Systematic text condensation was applied for analysis in order to uncover the concepts and themes. *Results*. The analysis revealed the following 3 main themes: (1) use of cardiovascular guidelines and risk assessment tools, (2) strategies for managing patient compliance, and (3) GPs' own risk management. There were substantial differences in the attitudes concerning all three themes. *Conclusions*. The substantial differences in the GPs' personal and professional risk perceptions may be a key to understanding why GPs do not always follow cardiovascular guidelines. The impact on daily clinical practice, personal consultation style, and patient behaviour with regard to prevention is worth studying further.

## 1. Introduction

Insight into general practitioners' (GPs) perception of risk is a cornerstone for understanding their strategies for practicing preventive care [1, 2]. The way persons perceive risk can be seen as part of a general personality trait, and healthcare professionals' risk perception has impact on their clinical decision-making [3–5]. Risk perception is influenced by a mixture of individual considerations, social conditions, and the specific context [6].

Lipid-lowering drugs are examples of preventive drugs that can reduce patients' cardiovascular risk, and specific clinical guidelines and risk assessment tools and charts have been developed for use in general practice [7]. However, differences exist between GPs' prescribing patterns regarding lipid-lowering drugs and clinical cardiovascular guidelines [8–10]. Moreover, patients' compliance is often low, as patients do not redeem their prescriptions or do not take their medication as prescribed [11, 12]. Patient compliance is an expression of the degree to which the patient decides to follow a treatment as agreed with the doctor. In some cases, compliance, adherence, and concordance are perceived as synonyms; in other cases, the concepts reflect differences in the doctor-patient relationship and the decision-making process. Patient compliance has proved to be particularly low for preventive treatment and for conditions that do not cause symptoms. Dyslipidaemia itself is not associated with symptoms, and the reduced blood lipids due to the effect of lipid-lowering drugs cannot be felt by the patient. The GP must consider the beneficial effect against the risk of side effects and take into account the patient's comedication, comorbidity, and life expectancy, as well as other patient factors, preferences, and the cost of the drug [13, 14]. GP risk perception can be associated with a broad spectrum of personal variables such as personality, communication style, and previous experiences, as well as demographic and organisational characteristics of the practice. The effect of the treatment on the individual patient's risk of developing cardiovascular disease has been shown to be difficult for the GP to explain to the patient and a challenge for the patient to understand [15–17]. Diversity in GPs' assessment of cardiovascular risk has been found [1, 18], but their views and considerations regarding managing dyslipidaemia and measurement of blood lipids, risk communication, and personal risk management have not previously been studied. The aims of this interview study were to explore GPs' professional and personal attitudes and experiences regarding cardiovascular guidelines and treatment with lipid-lowering drugs and their views on patient compliance in relation to medical understanding of risk.

## 2. Methods

In order to gain a deep insight into the GPs' perceptions of cardiovascular risk and compliance, we conducted a qualitative study based on semistructured individual interviews with ten GPs from general practices in the capital and southern region of Denmark with a combined population of approximately 2.9 million inhabitants, 1900 GPs, and 1140 practices.

### 2.1. Sampling

The material was drawn from qualitative in-depth interviews conducted by the first author, a medical doctor and Ph.D. student trained for the conduction of qualitative interviews and completing a comprehensive course in qualitative research methodology and interview technique offered by academic experts in social science and anthropology. Further, all interviews were carefully supervised by the second and last authors, both experienced qualitative researchers. We used purposive sampling, where participants are sampled according to preselected criteria relevant to the particular research question [19], in this case both demographic and professional characteristics: we identified 12 GPs from practices considering the GPs' age and gender and their association with single-handed practices, partnership practices, and practices from rural or urban areas. These characteristics are known to have an impact on the management of care and risk perception [3–5]. Thus, the sample represents experiences from all professional stages of a GP career from the young and newly established GP to the GP with more than 30 years of experience, GPs from urban and remote areas, female and male GPs, and so forth. The 12 GPs were approached: two of the 12 approached GPs did not respond to our contact and 10 were recruited and interviewed (Table 1). After 8 interviews no new themes seemed to emerge and data saturation was assumed to be reached. However, we chose to carry out the 2 last interviews as initially planned.

### 2.2. Interview Procedure

The interview guide was developed on the basis of a literature review and discussions in the research group comprising clinical pharmacologists, GPs, experts on medical risk analysis, and experienced qualitative researchers. The semistructured and iterative technique allowed participant responses to affect how and which questions the interviewer asked next. In that way emerging themes and perspectives were explored in the interviews with subsequent participants [20]. The following 4 main topics were covered in the interviews: management of dyslipidaemia and measurement of blood lipids, risk communication, pharmacological prevention, and personal experiences with cardiovascular risk; see Table 2.

The interviews were carried out from June to November 2013 in the GPs' practices and took 30–60 minutes each. The interviewer presented herself as a future GP and researcher and informed the GPs that they would remain anonymous and that the interview was part of a larger study on risk perception and compliance in the research portfolio of the Research Unit of General Practice. The GPs were encouraged to describe examples from their own practices and reflect on them and were informed that the focus was their personal attitudes and experiences rather than their knowledge of recommendations and guidelines. The second and the last author supervised all interviews.

### 2.3. Analysis

The ten interviews were digitally recorded and transcribed verbatim by the first author, and all authors collaborated on the analysis. In order to systematically uncover important themes and to get a rich straightforward description of the concepts and latent variables, the explorative approach of systematic text condensation was applied [21, 22]. The transcripts were read thoroughly to get an overall impression of the material before the initial coding. Then, meaning units in each transcript were identified and the data were coded, sorted, and categorised into themes and subthemes by identifying similar expressions, patterns, and sequences. Data from each of the coded groups were condensed and summarised into generalised descriptions and concepts concerning views on cardiovascular risk and views on preventive care. Finally quotes were selected to illustrate each theme and its related subthemes. During the analytical process the extracted information was related to the full transcripts in order not to lose the original context [21, 23].

## 3. Results

The analysis of the ten transcripts revealed the following 3 main themes: (1) cardiovascular guidelines and risk assessment tools, (2) strategies for managing patient compliance, and (3) GPs' own risk management.  Table 3 and the following sections summarise the results from the study. Quotes from the GPs a written in italics below to illustrate some of the views.

### 3.1. Cardiovascular Guidelines and Risk Assessment Tools

The GPs found recommendations and guidelines helpful, but they chose to make their own medley composed by the patient's wishes, preferences, age and polypharmacy, and adverse effects of the treatment. Further, some GPs stated that the recommendations lacked different thresholds for different patient groups and described the conflicts between guidelines and the laboratories' thresholds for blood lipids as confusing.

The GPs expressed the following attitudes towards recommendations and thresholds: some found that the recommendations were helpful as a guide for managing treatment and stated that they followed them strictly. Others mentioned that aspects such as the patient's age and handling of polypharmacy might lead to deviations from the recommendations. Others again found guideline recommendations based on epidemiological studies difficult to use in a daily clinical context with real patients. These GPs were aware of the recommendations but did not use them actively, and some even felt that guidelines inhibit practising as a genuine GP. Some of the reluctance to follow guidelines and recommendations was explained by opposing messages in different recommendations and a lack of different thresholds for blood lipids for different groups of patients. Instead some GPs made up their own thresholds for initiating preventive treatment.

*“Well I just follow them (the recommendations). I think they are fine, and they help me. They help me in the sense that we have made a note to put in the record before the patient comes in…to remind me what questions I should be asking”*  (Male 64 years, partnership practice).

*“They (the patients) can be fitted so much into charts that there is no room for me to be a doctor to them. I find that a bit of a problem”*  (Male 65 years, partnership practice).

The GPs' attitudes towards and experiences with using risk assessment tools to assess patients' cardiovascular risk and subsequent risk communication varied. Some GPs did not use risk assessment tools at all and almost felt aversion to putting patients into charts; others ceased to use them, as the tools did not provide sufficient support in the communication. A third approach was using the tools solely for risk assessment without explicitly involving the patient in the communication and decision-making.

*“Yes, I have used that before, but actually don't use it any more…. I felt that when I used the percentages, I didn't feel it was very helpful. When I used the general scoring (SCORE) [24], I often thought they (the patients) weren't really in it/didn't fit into it”*  (Female 60 years, single-handed GP).

*“…I don't necessarily involve the patient in it, but it's just to find out…by myself, where we are”*  (Male 47 years, partnership practice).

### 3.2. Strategies for Managing Patient Compliance

The GPs' strategies for achieving good patient compliance, their communication style, inclination to check patients' prescription redemption, and reaction to low compliance varied. Also, the GPs' attitudes towards allocation of responsibility of treatment between the patient and the GP varied. Two different ways of managing compliance were identified: a confrontational and a resigned way. In the resigned way, two different views on where to place the responsibility for the treatment were represented: compliance was either seen as the patient's own choice, which should be respected and not questioned by the GP, or seen as no problem at all, since drug treatment is the GP's choice and the patient is expected to just comply. In the confrontational group two different strategies were represented: presenting the patient with different scenarios of cardiovascular events that might take place if the drug treatment is not followed and having repeated consultations to reveal barriers towards following the agreed treatment.

*“I am, after all, a rather paternalistic doctor, so the patients mostly do what I think and recommend they should do, without us getting into big and deep discussions”*  (Male 65 years, partnership practice).

*“If I feel that (The GP recounts a conversation between a patient and himself) Patient: ‘Really, doctor…', GP: ‘Well, okay. You can then see the nurse, or you can see me once a year. Then we don't need to…But you are welcome to come back, if you change your mind…and then we have to be a bit more intensive about your disease…For instance, if you had a stroke' ”*  (Male 64 years, partnership practice).

In Denmark all GPs are able to continuously monitor patients' redemption of prescriptions through a national website (https://www.sundhed.dk/). The questions of whether or not to monitor patients' prescription redemption showed considerable diversity: from GPs monitoring patients' redemptions regularly to GPs considering trust and reliance on the patient more important than control in order to make the best decision to GPs rejecting problems with patient compliance.

*“No, not at all, I don't access sundhed.dk, then I would feel that I am moving beyond…or at least it is not the type of doctor I want to be, being that way controlling”*  (Male 40 years, single-handed GP).

*“I know they do (redeem the prescription)! …So this compliance thing, that's not a problem, it really isn't...”*  (Male 65 years, single-handed GP).

The GPs' reactions to patients with low compliance varied from just accepting low compliance as the patient's choice to irritation to frustration over the time wasted on consultations and repeated prescription renewals.

*“Sometimes with surprise, sometimes with irritation, and other times I just think that it's her own problem”*  (Male 40 years, single-handed GP).

*“Then I get annoyed, because I feel it's a waste of my time, just as it is wasting my time to come for check-up for your diabetes or something else, if you don't take the medicine”*  (Female 56 years, single-handed GP).

### 3.3. GPs' Own Risk Management

The last theme concerned the GPs' personal risk management, their attitudes to measuring their own blood lipid level and taking lipid-lowering drugs themselves. The GPs' views on measuring their own blood lipid level varied: from some GPs having measured it if they had a clear indication to some having measured it several times out of curiosity without having a clear indication to others not knowing or caring about their own blood lipids. The analyses revealed that reluctance towards taking lipid-lowering drugs exists among GPs. It was expressed that it could be a big challenge recognising dyslipidaemia as a problem serious enough to be solved with drugs.

*“No way would I consider taking statins, because I would calculate the same way as for my patients that I have no reason to fear a cholesterol level of 7. So it's a bit on the same rational basis, I think”*  (Male 47 years, partnership practice).

*“I thought that I just needed to know it (the cholesterol level), well not because it worried me, but…2005…and then I have measured it 7 times since then, that may be over the top”*  (Male 65 years, partnership practice).

## 4. Discussion

This study provides insights to GPs management of cardiovascular risk both professionally and personally. It also provides answers to why GPs may not always follow guidelines for CVD prevention. Differences in their use of cardiovascular guidelines and attitudes to assessing and communicating cardiovascular risk play important roles, as well as their views of their own cardiovascular risk.

In the sampling procedure we aimed for a variety in relevant GP characteristics in order to obtain diversity in the views on risk perception. However, it cannot be ruled out that the GPs who agreed to participate may have a bigger interest in drugs and pharmacotherapy than Danish GPs in general and therefore our interviews may not have covered the whole spectrum of views.

The use of only one interviewer may imply both strengths and weaknesses related to the interviewer's preconceptions and special interest in views on cardiovascular risk.

The dialogue with a colleague, the promised anonymity, and carrying out the interviews in the GP's own clinic may have ensured openness and confidentiality. However, some degree of perceptiveness in the meeting between two clinicians could probably not be avoided [25]. The role of the interviewer was minimized by broad and critical reading within the research group consisting of multiple researches with different backgrounds, that is, clinical pharmacologists, GPs, and experts on medical risk analysis. Here preconceptions were shared and discussed, and supplementing and contesting each other's statements established important metapositions [22]. This increased the understanding of the complex phenomena of risk and compliance.

Asking clinicians directly about their professional work and attitudes towards recommendations and prescribing practices may have prompted some GPs to give “correct” and normative answers, more in accordance with clinical recommendations and guidelines than with their actual practices and thereby reducing validity [26, 27]. The GPs were therefore also asked to talk about the most recent patient they treated with lipid-lowering drugs (Table 2). In this way the perspectives and approaches towards cardiovascular risk management were probably concretised and nuanced.

Different approaches to application and implementation of cardiovascular guidelines were found in the data. From GPs finding guidelines good and applicable to GPs finding the process from epidemiological studies and patients in flesh and blood challenging since it requires too much tailoring to fit the needs of the individual patient. Other GPs find that guidelines inhibit the ability to practice independently as a GP and practice patient centered care. This is in line with Backlund et al. who found that some GPs explain guideline deviation with guidelines being too simple in some aspects [28]. Graversen et al. [9] stated that the gap between recommended lipid-lowering drug therapy and current practice causes substantial undertreatment and considerable delay in the first prescription of lipid-lowering drugs. Both rational and irrational choices were mentioned as causes for not following guidelines [29, 30]. Hetlevik et al. [31] pin that GPs who do not follow practice guidelines may have good reasons and that it might be an expression of civil disobedience among particularly ambitious doctors with the best intentions calling for a reassessment of the evidence that guidelines are based on. Others state that the most important causes for not following guidelines are unsatisfactory knowledge of guidelines and barriers to the process of changing clinical practice in order to implement guidelines. Subjective factors and individual professional characteristics such as awareness, knowledge, attitude, motivation to change, and behavioural mechanisms play an important role [3, 32]. Further, the use of risk assessment tools and guidelines as well as clinicians' actual prescribing behaviour may be influenced by such subjective factors [33]. Future research in concrete actions to facilitate the transition from clinical guidelines to daily clinical practice should not only take external and organisational factors into account but also consider differences in GPs subjective factors and personality traits.

Different approaches also existed in the strategies to manage patient compliance with statins. Quite strikingly, some GPs in the study did not recognise compliance as a potential issue in clinical practice and expected patients to simply comply with their prescriptions. Others reported to be very concerned about enhancing compliance and revealing potential barriers. It seemed that younger GPs were more concerned with enhancing patient compliance than older GPs. This continuum of approaches might be related both to differences in personal communication style and to the paradigm shift from the classic notion of a paternalistic doctor-patient relationship to one of shared decision-making and informed consent—a mutual interactive process between the doctor and the patient who jointly make a health decision [34]. Although different models to enhance patient compliance have been suggested [35, 36], the most efficient way for the GP to address patient compliance is still not fully understood.

The analysis in the present study revealed reluctance among some GPs towards taking lipid-lowering drugs themselves. Yet no informants described problems prescribing these drugs to their patients and putting effort into enhancing patient compliance. One of our preconceptions was that the GPs' personal risk perception is associated with their patients' behaviour in relation to prevention. Some might fail to see the relevance of GPs taking blood samples or even statins themselves to be a measure of how they treat their patients with elevated blood lipids. It has, however, previously been shown that doctors personal health habits and beliefs about counselling [37, 38], as well as personal characteristics [39, 40], strongly influence their practices in counselling patients about health habits. We recognise that a GP's prescribing decision is also the result of many external inputs from the patient, commercial sources, professional colleagues, the academic literature, and government regulators [41]. Gale et al. found striking disconcordance between some GPs' prescribing of preventive cardiovascular medication for their patients and preference of lifestyle changes for themselves [42]. This is in line with the GPs in the present study expressing that they largely refrain from following the advice on lifestyle they give their patients. Do the GPs believe that they are less at risk of cardiovascular disease than their patients, even if they have the same blood cholesterol level? Might this be an example of optimistic bias that the GPs and patients have in common explaining some of the problems with compliance [43]? The different professional and personal approaches to prevention among some GPs might also be a sign of the very act of making a recommendation change the ways GPs think in relation to medical choice as proposed by Ubel et al. [44].

An analysis of the association between GPs' professional and personal views on cardiovascular risk and their actual behaviour, for example, regarding prescribing patterns for lipid-lowering drugs, might be obtained through a quantitative approach using questionnaires in combination with registers on prescribed and redeemed medication.

## 5. Conclusion

The substantial differences in the GPs' personal and professional risk perceptions may be a key to understanding why GPs do not always follow cardiovascular guidelines. The impact on daily clinical practice, personal consultation style, and patient behaviour with regard to prevention is worth studying further.

## Figures and Tables

**Table 1:** GP characteristics.

Gender	Female	4
	Male	6

Age	<50 years	3
	≥50 years	7

Practice organisation	Single-handed GPs	5
	Partnership practices	5

Patient recruitment area	Rural	4
	Urban	6

**Table 2:** Interview guide, probing questions.

Main topics	Probing questions
Management of dyslipidaemia and taking blood lipids	Please tell me about the latest patient you started on lipid-lowering treatment here in the clinic

Risk communication	(i) What is most important to you when you talk to a patient about cardiovascular risk? (ii) How do you use risk assessment tools?

Pharmacological prevention	How much importance do you attach to treatment with lipid-lowering drugs?

Personal experiences with cardiovascular risk	(i) Do you know your own cholesterol level? (ii) How do you manage your own cardiovascular risk?

**Table 3:** Summary of results.

Themes	Related subthemes	Differences in attitudes
Cardiovascular guidelines and risk assessment tools	Cardiovascular guidelines	(A) Good and applicable, follow them virtually completely (B) The difference between the academic approach from epidemiological studies and patients in flesh and blood is challenging (C) Cardiovascular guidelines inhibit the ability to practice independently as a GP
	Use of risk communication tools	(A) Not sufficient power in the risk communication (B) Only for risk assessment, not for risk communication (C) Thorough use of different tools depending on the patient context

Strategies for managing patient compliance	Resigned approach	(A) Prescribing is the GP's choice and patients comply (B) Taking preventive drugs is solely the patient's choice
	Confrontational approach	(A) Describing consequences of low compliance (B) Revealing barriers to low compliance

Personal risk management	Measuring own blood lipids	(A) Yes, with clear indication (B) Yes, without clear indication (C) No, never
	Using lipid-lowering drug themselves	(A) Reluctant (B) Pragmatic
